# Direct Intraocular Lens Extraction Using a Newly Developed Lens-Grabbing Forceps

**DOI:** 10.3390/jcm13102938

**Published:** 2024-05-16

**Authors:** Santaro Noguchi, Shunsuke Nakakura, Hitoshi Tabuchi, Asuka Noguchi

**Affiliations:** 1 Department of Ophthalmology, Saneikai Tsukazaki Hospital, Himeji 671-1227, Japan; s.nakakura@tsukazaki-eye.net (S.N.); h.tabuchi@tsukazaki-eye.net (H.T.); 2 ASUCA Eye Clinic, Sendai 980-0021, Japan; a-k2005@hotmail.co.jp

**Keywords:** intraocular lens extraction, lens dislocation, cataract

## Abstract

**Background:** Due to lower age thresholds for cataract surgery and increased longevity, cases with intraocular lens (IOL) dislocation requiring removal have increased. Traditional methods, such as cutting or folding the IOL within the eye, pose a high risk of complications, including corneal endothelial and iris damage. **Methods:** We developed a new minimally invasive technique for direct IOL removal using specially designed lens-grabbing forceps. These forceps can grasp and remove the IOL through a small incision in a single motion, significantly reducing intraocular manipulations compared to conventional methods. **Results:** In our test cases, IOL removal through a 2.2 mm corneal incision was completed in approximately 95 s, with minimal incision enlargement (about 0.16 mm) and a slight decrease in corneal endothelial cells. **Conclusions:** Our findings suggest that this technique is minimally invasive and safe for IOL removal, offering a promising alternative to existing methods.

## 1. Introduction

Currently, most cases requiring intraocular lens (IOL) removal involve posterior chamber IOLs. The most common reasons for IOL removal include exchanging an incorrectly powered IOL [1,2], patient dissatisfaction due to refractive surprises after surgery [3], dysphotopsia or photic phenomena caused by multifocal IOLs [4,5,6], opacification of acrylic IOLs [7], and IOL dislocation in eyes with weak zonules [8,9,10].

Currently available removal techniques for foldable IOLs include cutting the IOL into pieces inside the eye or folding it to allow removal through a small incision [11,12,13,14,15] Considering that most implanted IOLs nowadays are soft foldable lenses, the split and cut technique through a small incision has been used quite frequently [11,12]. However, excessive intraocular manipulation can damage the posterior capsule and corneal endothelium [16,17,18]. The cartridge removal technique has also been reported more recently as an alternate approach to minimizing intraocular manipulation and reducing the risk of corneal endothelial damage and posterior capsular rupture [19]. These techniques are designed for the removal of acrylic IOLs. Neither method of IOL removal is simple. Conventional techniques can cause damage to the intraocular tissues. Also, in cases where there is no posterior capsule, the IOL can easily fall into the vitreous cavity during the surgical procedure using conventional techniques.

In this study, we introduce a newly developed technique and specially designed forceps for the direct removal of IOL through a small incision. We also present the initial clinical results in 10 consecutive eyes.

**Figure 1:**
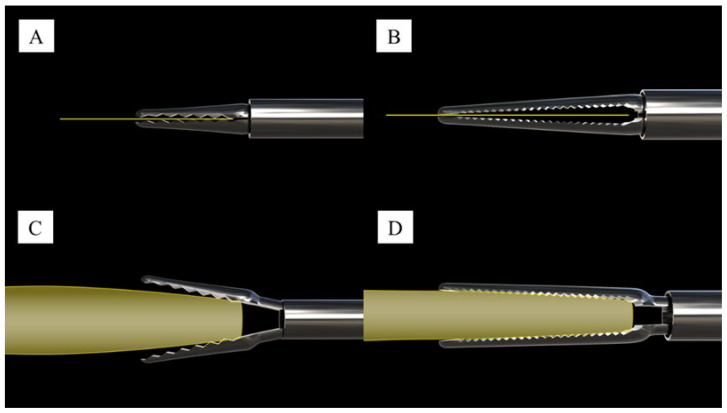
Conventional versus newly developed lens-grabbing forceps. (**A**): Conventional forceps grasping a thin object. (**B**): Novel lens-grabbing forceps grasping a thin object. (**C**): Conventional forceps holding an intraocular lens (IOL). (**D**): Novel lens-grabbing forceps holding an IOL.

We designed lens-grabbing forceps (Inami, product number DS-2022L) to grasp the IOL inside the eye firmly. Although conventional forceps can grasp thin or slender objects, they cannot firmly grasp thicker objects along their whole length. The new IOL removal forceps are designed to grasp thick objects such as IOLs along a wide area and their entire length, allowing the clinician to evenly grasp the optic surface (Figure 1). After the foldable IOL is grasped with the forceps, it is pulled toward the incision. Near the incision, the IOL deforms in the pulling direction and naturally rolls up, fitting into the incision and slowly prolapsing out.

## 2. Materials and Methods

We determined the feasibility of direct IOL extraction using a lens-grabber on pig eyes, examining from the time the IOL was placed on the iris to the time it was completely extracted from the eye. The IOLs (AcrySof, Alcon, Fort Worth, TX, USA) were extracted while confirming the following observation points: the ability of the IOLs to be extracted using a lens-grabber, the shape into which the IOLs deformed during the process of extraction, contact between the IOLs and the corneal endothelium or iris during the extraction process, and damage of the extracted IOLs. The removal of the IOLs from within the eye was observed using a lacrimal canal endoscope.

After confirming the safety and feasibility of extracting the IOLs from the pig’s eye, the actual surgical extraction was examined. The extracted IOL was an AcrySof. In human eye, the safety ware confirmed too with this IOL (AcrySof) removal technique using a lens-grabber.

The clinical results of direct IOL extraction using a lens-grabber were reviewed in several cases. Patients who underwent IOL extraction with this new technique at Saneikai Tsukazaki Hospital (Himeji, Japan) from April 2021 to May 2022 were included. The research outline was posted on the bulletin board and website of Saneikai Tsukazaki Hospital and announced to eligible patients. In all cases, a 2.2 mm clear corneal incision was made, and the IOL was removed using the lens-grabbing forceps (Inami, Tokyo, Japan) without cutting the IOL. The time taken for IOL removal, the change in corneal endothelial cell count from before surgery to 3 months postoperatively, the incision size enlargement, the need for wound suturing, iris damage, and other complications were evaluated. The incision size was measured using an incision gauge (ME Technica, Tokyo, Japan) immediately after the incision was made and after IOL removal. Incision size was defined as the gauge size that just passed through the wound. Removal time was defined as the time taken from grasping the IOL in the anterior chamber until complete removal.

## 3. Results

Our findings showed that direct IOL extraction from a pig’s eye using a lens-grabber was feasible. The extracted IOL deformed circularly into a rolled shape with the corneal side convex, fit into the incision, and was extracted. The IOL was removed without damaging the cornea or iris.

**Figure 2:**
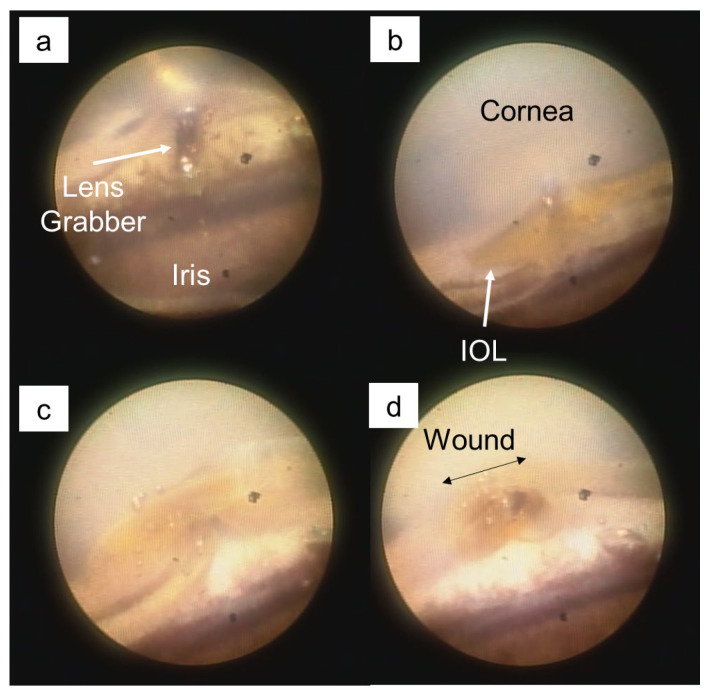
Intraocular lens (IOL) removal in porcine eyes. (**a**): IOL grasped using lens-grabbing forceps. (**b**): IOL entering the incision incision. (**c**): One-third of the IOL in the incision. (**d**): Two-thirds of the IOL are grasped using lens-grabbing forceps.

Figure 2 shows the extraction of an SN60WF (AcrySof, Alcon, Fort Worth, TX, USA) IOL in porcine eyes through a 2.2 mm limbal tunnel incision using the new technique via a lacrimal endoscope. It can be observed that the IOL naturally rolled along the incision and could be extracted without touching the cornea or iris. Similarly, in human eyes, the IOL is extracted through a mountain fold pattern toward the surgeon.

**Figure 3:**
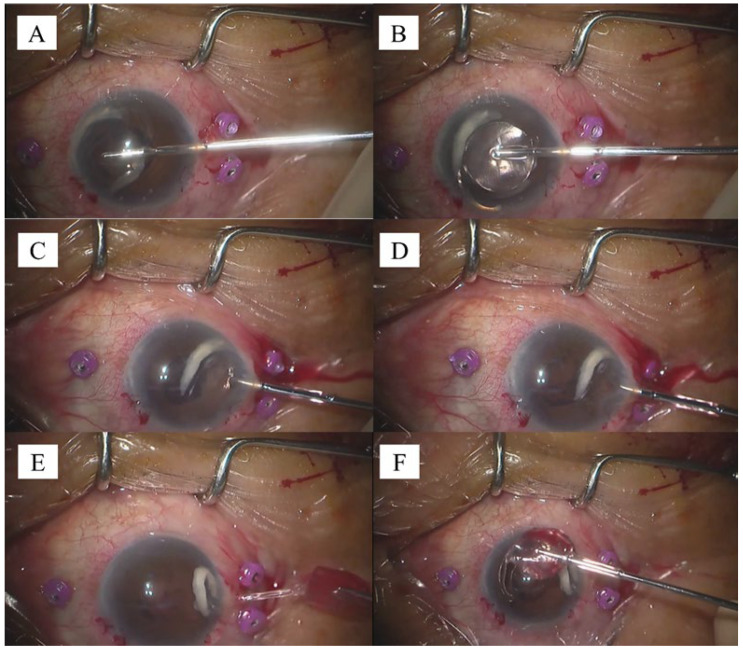
Hydrophobic intraocular lens (IOL) removal using the lens-grabbing forceps. (**A**): IOL placed on the iris with the haptic and optic junction positioned near the incision. Haptics grasped firmly using the forceps. (**B**): Haptics and the center of the optic surface grasped firmly. Adequate viscoelastic material behind the IOL to prevent iris involvement. (**C**): IOL rolling up into a mountain fold while being pulled out of the incision. (**D**): IOL slowly being pulled through the incision parallel to the incision direction. (**E**): Full prolapse of the IOL out of the incision. (**F**): Removed IOL inspected for any damage or retained fragments.

The IOL could also be directly extracted from human eyes. Details regarding the extraction method are as follows: Initially, a 2.2 mm corneal incision is made at the limbus. The acrylic IOL to be removed is then placed on the iris, after which the anterior chamber is filled with viscoelastic material warmed to approximately 37 °C to keep the IOL soft. The IOL, including the haptic junction, is grasped firmly with the forceps (Figure 3A,B) and then slowly pulled toward the incision. The pulling direction should be aligned with the incision to prevent iris damage when the IOL rolls up (Figure 3C). Thereafter, the IOL is slowly pulled further out of the incision. With gentle traction, the IOL naturally rolls up from its stretched state and is extracted out of the eye (Figure 3D). Once fully removed (Figure 3E), the IOL is examined for any damage or retained fragments (Figure 3F and Video S1).

### Clinical Results with Lens-Grabbers for 10 Consecutive Cases

The mean IOL removal time was 94.5 ± 21.2 s. The mean corneal endothelial cell loss was 293 ± 114 cells (11.2 ± 3.7% decrease). The pre- and post-removal incision size was 2.17 ± 0.08 (range 2.1–2.3) mm and 2.33 ± 0.07 (2.2–2.4) mm, respectively, with a mean enlargement of 0.16 ± 0.05 (0.1–0.2) mm (Table 1). None of the included cases developed intraoperative complications. Wound closure via cornea stromal hydration was adequate in all cases without suturing. No iris damage was observed. All IOLs successfully prolapsed out with a mountain fold pattern and with no tears or damage.

## 4. Discussion

In this study, we introduce a new direct IOL removal technique using the newly developed lens-grabbing forceps. The use of the lens-grabbing forceps in all 10 cases resulted in a mean IOL removal time of approximately 95 s, with a mean corneal endothelial cell loss of approximately 290 and a mean incision enlargement of only 0.16 mm. Overall, the lens-grabbing technique enabled quick IOL removal with minimal intraocular damage and wound enlargement.

The degree of incision enlargement likely depends on the rigidity of the IOL. However, Lens-grabbar technique with comparable softness IOL to those studied herein is expected to produce similar outcomes to minimal incision enlargement. Although the current study evaluated nine hydrophobic and one hydrophilic IOLs, similar results are expected with hydrophilic acrylic three-piece IOLs. The lens-grabbing forceps are designed to grasp the entire IOL optic uniformly regardless of lens thickness and configuration. The IOL in the eye is warmed by body temperature; however, if the Visco to be inserted into the eye is cold, the IOL temperature will decrease and the IOL may become stiff, so the Visco should be left warmed to the same degree as body temperature. This enables single-action removal through small incisions of <2.2 mm, which is not possible using conventional forceps. This novel technique can be used with any soft IOL, including silicone IOL. In the case of silicone IOLs, it is also useful to use the forceps with the other hand to pull out the IOL together during extraction. In the case of a three-piece IOL, if the haptics are made of a hard material, such as PMMA, it is necessary to make sure that all of the haptics are removed, as they may break (Video S2).

Previously reported techniques involve cutting the IOL inside the eye [11,12,13,14,15] or using a cartridge [19]; however, these techniques involve more intraocular manipulation. Our newly developed technique and forceps presented herein minimize manipulation and enable rapid and safe IOL removal. Our findings confirmed that our technique and forceps promoted minimal wound enlargement (0.16 mm) and endothelial cell loss, highlighting the several advantages of our technique over conventional methods. The IOL extraction technique we report here is the least intraocularly demanding, requiring only forceps to grasp the IOL within the eye and pull it out. Conventional techniques require several intraocular steps involving both hands. The simplicity of the procedure may have resulted in a very short surgical time for removal.

Although the cartridge technique for IOL removal reported by Fukuoka et al. [19] also avoids intraocular cutting, their technique required a cartridge, insertion into the eye, and two-handed maneuvers. Our forceps technique is simpler and enables one-handed surgery, leaving the other hand free. Clinical incision enlargement had not been reported using the cartridge technique but is expected to be greater than that using our forceps technique.

In this study, a 0.16 mm wound expansion was observed at a 2.2 mm incision, but the 2.4 mm incision was not studied in detail. If this procedure is performed with a 2.4 mm incision and the wound is not enlarged, it may be possible to consider performing the procedure with a 2.4 mm incision from the beginning.

One possible complication of this new technique is that, when the IOL is rolled and removed from the eye, if not enough Visco is filled over the iris and other areas, it may engulf the iris and result in iris damage, which could be a complication. In addition, the old surgical wound overlaps the extraction wound, and the fragility of the wound may lead to wound enlargement and failure of wound closure.

## 5. Conclusions

We developed a novel technique and lens-grabbing forceps that allow for direct IOL removal through a small incision. In all 10 cases examined, the mean IOL removal time was 95 s, with a mean incision enlargement of 0.16 mm and minimal corneal endothelial cell loss. Our findings suggest that the developed technique was minimally invasive and safe for IOL removal. Slight differences in incision enlargement may occur depending on the power and type of IOL to be removed, and this aspect warrants further investigation.

## Figures and Tables

**Table 1:** Patient characteristics and postoperative results.

Case No.	Age	R or L	Surgery Methods	Extraction IOL	Extraction IOL Power	Fixed IOL	IOL Power	Axial Length	K1	K2	Pre-op Endothelial Cell	Post-op Endothelial Cell	Pre-Wound Size (mm)	Post-Wound Size (mm)
1	79	R	IOL exchange	MINIWELL TORIC	21	NX-60	19	23.1	7.47	6.98	3018	2531	2.1	2.3
2	51	R	IOL exchange	unknown	unknown	NX-60	18	24.93	8.58	7.29	2234	1853	2.1	2.2
3	64	R	27GVit + IOL intrascleral fixation	ZLB00	15	NX-60	16	25.9	7.92	7.78	2330	2190	2.2	2.4
4	77	R	27GVit + IOL intrascleral fixation	ZCB00	18	NX-60	18	25.2	7.35	7.21	2440	2150	2.2	2.4
5	65	L	27GVit + IOL intrascleral fixation	ZXR00V	15	NX-60	16	25.85	7.89	7.79	2564	2220	2.3	2.4
6	83	R	27GVit + IOL intrascleral fixation	SN60WF	unknown	NX-60	15.5	25.96	8	7.63	2450	2160	2.2	2.3
7	56	R	IOL exchange	DIB00V	15	LS-313 MF15T2	13	26.99	8.01	7.83	3157	2838	2.3	2.4
8	56	L	IOL exchange	DIB00V	16	LS-313 MF15T1	14.5	26.89	8.12	7.96	2940	2670	2.1	2.3
9	78	R	IOL exchange	LS-313 MF15	24	NX-60	25	21.27	7.16	7.05	2956	2626	2.1	2.3
10	52	L	27GVit + IOL exchange	DIB00V	24	NX-60	24.5	22.55	7.89	7.6	1436	1350	2.1	2.3

MINIWELL TORIC (SIFI MedTech, Catania, Italy), ZLB00 (Johnson & Johnson Vision, Inc., Irvine, CA, USA), ZCB00 (Johnson & Johnson Vision, Inc., Irvine, CA, USA), ZXR00V (Johnson & Johnson Vision, Inc., Irvine, CA, USA), DIB00V (Johnson & Johnson Vision, Inc., Irvine, CA, USA), LS-313 MF15, LS-313 MF1T1 and T2 (Santen, Osaka, Japan), NX-60 (Santen, Osaka, Japan).

## Data Availability

The datasets generated and/or analyzed during the current study are available from the corresponding authors on reasonable request.

## Supplementary Materials

The following supporting information can be downloaded at: https://www.mdpi.com/article/10.3390/jcm13102938/s1, Video S1: Direct extraction of hydrophobic acrylic IOL using a lens-grabber; Video S2: Direct removal of silicone IOL using lens-grabber.

## References

[1] Jin G.J., Crandall A.S., Jones J.J. (2005). Changing indications for and improving outcomes of intraocular lens exchange. Am. J. Ophthalmol..

[2] Mamalis N., Brubaker J., Davis D., Espandar L., Werner L. (2008). Complications of foldable intraocular lenses requiring explantation or secondary intervention—2007 survey update. J. Cataract Refract. Surg..

[3] Mavroforou A., Michalodimitrakis E. (2003). Physicians’ liability in ophthalmology practice. Acta Ophthalmol. Scand..

[4] Galor A., Gonzalez M., Goldman D., O’Brien T.P. (2009). Intraocular lens exchange surgery in dissatisfied patients with refractive intraocular lenses. J. Cataract Refract. Surg..

[5] Woodward M.A., Randleman J.B., Stulting R.D. (2009). Dissatisfaction after multifocal intraocular lens implantation. J. Cataract Refract. Surg..

[6] de Vries N.E., Webers C.A., Touwslager W.R., Bauer N.J., de Brabander J., Berendschot T.T., Nuijts R.M. (2011). Dissatisfaction after implantation of multifocal intraocular lenses. J. Cataract Refract. Surg..

[7] Lane S.S. (2004). Foldable intraocular lens removal/exchange: Can it be prevented?. Ophthalmology.

[8] Jones J.J., Jones Y.J., Jin G.J. (2014). Indications and outcomes of intraocular lens exchange during a recent 5-year period. Am. J. Ophthalmol..

[9] Leysen I., Bartholomeeusen E., Coeckelbergh T., Tassignon M.J. (2009). Surgical outcomes of intraocular lens exchange: Five-year study. J. Cataract Refract. Surg..

[10] Fernández-Buenaga R., Alió J.L., Pérez-Ardoy A.L., Larrosa-Quesada A., Pinilla-Cortés L., Barraquer R., Alio J.L., Muñoz-Negrete F.J. (2013). Late in-the-bag intraocular lens dislocation requiring explantation: Risk factors and outcomes. Eye.

[11] Karamaounas N., Kourkoutas D., Prekates C. (2009). Surgical technique for small-incision intraocular lens exchange. J. Cataract Refract. Surg..

[12] Mehta J.S., Wilkins M.R., Gartry D.S. (2005). Explantation of an acrylic Acrysoft intraocular lens without wound enlargement. Acta Ophthalmol. Scand..

[13] Por Y.M., Chee S.P. (2007). Trisection technique: A 2-snip approach to intraocular lens explantation. J. Cataract Refract. Surg..

[14] Osher R.H. (2006). Crisscross lensotomy: New explantation technique. J. Cataract Refract. Surg..

[15] Narang P., Steinert R., Little B., Agarwal A. (2014). Intraocular lens scaffold to facilitate intraocular lens exchange. J. Cataract Refract. Surg..

[16] Lee S.J., Sun H.J., Choi K.S., Park S.H. (2009). Intraocular lens exchange with removal of the optic only. J. Cataract Refract. Surg..

[17] Theoulakis P.E., Brinkmann C.K., Petropoulos I.K., Gatzogias M.I., Katsimpris J.M. (2009). Hydrogel intraocular lens exchange: Five-year experience. Klin. Monatsblätter Augenheilkd..

[18] Kubaloglu A., Sari E.S., Koytak A., Cinar Y., Erol K., Ozerturk Y. (2011). Intraocular lens exchange through a 3.2-mm corneal incision for opacified intraocular lenses. Indian J. Ophthalmol..

[19] Fukuoka S., Kinoshita T., Morita S., Sakurai T. (2021). Intraocular lens extraction using the cartridge pull-through technique. J. Cataract Refract. Surg..

